# “God gives the child”: An abductive analysis of barriers to postnatal care using the Health Equity Implementation Framework

**DOI:** 10.1177/17455057261424102

**Published:** 2026-04-06

**Authors:** Emilie Egger, Befikadu Bitewulign, Humberto Gonzalez Rodriguez, Haley Case, Abiyou Kiflie Alemayehu, Elizabeth C. Rhodes, Abiy Seifu Estifanos, Kavita Singh, Dorka Woldesenbet Keraga, Marukh Zahid, Hema Magge, Dara Gleeson, Clare Barrington, Ashley Hagaman

**Affiliations:** 1 Department of Psychiatry, Perelman School of Medicine, University of Pennsylvania, Philadelphia, PA, USA; 2 Department of Social and Behavioral Sciences, Yale School of Public Health, Yale University, New Haven, CT, USA; 3 Center for Methods in Implementation and Prevention Sciences, Yale University, New Haven, CT, USA; 4 Institute for Healthcare Improvement, Addis Ababa, Ethiopia; 5 Department of Health Behavior, Gillings School of Global Public Health, University of North Carolina at Chapel Hill, NC, USA; 6 CDC Foundation, Atlanta, GA, USA; 7 Hubert Department of Global Health, Rollins School of Public Health, Emory University, Atlanta, GA, USA; 8 Center for Implementation Sciences (CIS) in Health, School of Public Health, Addis Ababa University, Ethiopia; 9 Carolina Population Center, University of North Carolina at Chapel Hill, NC, USA; 10 Department of Maternal and Child Health, Gillings School of Global Public Health, University of North Carolina at Chapel Hill, NC, USA; 11 Department of Reproductive, Family, and Population Health, School of Public Health, Addis Ababa University, Ethiopia; 12 Yale School of Public Health, Yale University, New Haven, CT, USA; 13 Bill & Melinda Gates Foundation, Seattle, WA, USA

**Keywords:** maternal health, implementation, postnatal care, qualitative, abductive, Ethiopia

## Abstract

**Background:**

Postnatal care (PNC) is recommended as a means of preventing maternal mortality during the postpartum period, but many women in low- and middle-income countries do not access care during this period.

**Objective:**

We set out to examine sociocultural preferences that have been portrayed as barriers to care.

**Design:**

We designed a qualitative study using the Health Equity Implementation Framework (HEIF).

**Methods:**

We performed an abductive analysis of 63 semi-structured interviews with women who had recently given birth in three regions of Ethiopia using the HEIF and an inductive–deductive codebook to understand why women in Ethiopia do not use recommended PNC.

**Results:**

We found that, in many cases, health providers do not consider women’s cultural safety a primary need, but rather as a barrier to care. However, women’s perceived refusal to participate in postnatal visits was, for many, an expression of agency and assertion of their needs for cultural safety.

**Conclusion:**

We propose adding cultural safety to HEIF as a process outcome so that implementers consider cultural needs in a dynamic manner that does not ask patients to choose between meeting their cultural needs and receiving necessary health care during the postnatal period.

## Plain language summary

Understanding the importance of cultural safety to health equity and postnatal care uptake

This article illustrates how patients find it difficult to maintain their cultural safety during postnatal care appointments. While this is sometimes characterized by health providers as refusal to participate in health treatment, patients often consider it an assertive expression of their own health priorities. Our article demonstrates that categorizing cultural factors as “barriers to care” is not useful to successfully implementing evidence-based interventions

## Contributions to the literature

- This article illustrates how end users encounter interventions while maintaining their cultural safety.
- Our study highlights how end user agency is present at all stages of implementation and extends beyond the simple choice of whether or not to engage an intervention.
- Our article demonstrates that categorizing cultural factors as “barriers to care” is not useful to successfully implementing evidence-based interventions.

## Background

### The role of cultural safety in understanding barriers and facilitators to Postnatal care in Ethiopia

Maternal mortality has fallen over the past several decades.^1^ However, maternal morbidity and mortality remain very high during the postpartum period, defined as the first 6 weeks following birth.^2^ Globally, most maternal deaths occur during the postpartum period.^3^ Low- and middle-income countries (LMICs) have higher rates of maternal mortality than high-income countries.^4^ In LMICs nearly half of maternal deaths occur within 1 day of delivery and one-fifth during days 2–7 following delivery.^5^ In Ethiopia, intrapartum and postpartum deaths account for the majority of maternal mortality.^6^ In Ethiopia, a high number of maternal deaths occur within 48 hours of birth.^7^ While maternal morbidity is difficult to calculate, rates are higher in LMICs than in high-income countries.^8^ To address maternal morbidity and mortality, women need access to high-quality postnatal care (PNC).

PNC is the evidence-based intervention that forms the basis for our implementation study. A systematic review of the literature states that home visits are effective in preventing maternal and neonatal deaths.^9^ Several randomized controlled trials have shown that home visits by community health workers are effective at reducing perinatal mortality in resource-limited settings.^10–12^ Evidence from three randomized controlled trials indicated that these visits lowered perinatal mortality by 18%.^13^ Postnatal visits assess many aspects of maternal well-being, such as hypertensive status, psychological wellness, perineal wounds, status of lochia, infant feeding issues, and other critical health issues in the postpartum patient.^2^

The 2018 Ethiopian National Guidelines on PNC recommended that patients stay in the hospital or health center for at least 24 h after giving birth.^7^ For healthy women, the World Health Organization recommends a minimum of four PNC contacts, to be conducted within 24 h after birth, between 48 and 72 h after birth, between 7 and 14 days after birth, and during the sixth week after birth.^2^ The Ethiopian Ministry of Health set a goal of increasing the percentage of national adherence to 82% by 2025 and to increase the number of women who stay in the hospital or health center for 24 h after giving birth by 25%.^6^ Much of this work will be carried out on the community level and will involve the influence of community and religious leaders.^3^ PNC visits in Ethiopia are typically performed by female health extension workers (HEWs) at the patient’s home or at her local health post.^14^

### Literature review

The Ethiopian Ministry of Health has provided guidelines that state women who deliver at home should receive three postnatal visits, while for institutional deliveries, two PNC visits should commence on day 2 (48–72 h), as the first PNC visit will be given within the first 24 h of PNC in the health institution of delivery. HEWs perform all home visits.^14^ These visits are performed with infant neonatal and well-baby visits. Recent data have shown that the incorporation of HEWs has had an insignificant effect on increasing PNC visits.^15^

However, according to a recent scoping review, in LMICs, PNC was the least utilized service across the continuum of maternal care, with the largest drop off in care coming after institutional births.^16^ In Ethiopia, only 34% of women had a postnatal examination 2 days after birth.^17^ Nearly one half of women who delivered their babies in hospitals or health centers did not receive their first PNC exam.^17^ Despite efforts to increase PNC^18^ and much quantitative data on facilitators and barriers to care,^19–21^ little is known about the role of cultural safety in accessing PNC in these settings.^22^

Cultural safety is a concept developed by Māori nurses in New Zealand that addresses the relationship between patients’ cultural needs being met alongside positive health outcomes.^23–25^ Community and cultural considerations are at the core of cultural safety. For example, perinatal patients reported that kinship networks were critical to achieving cultural safety.^26^ This framework reframes previously reported barriers to PNC care by underscoring how individuals enact their agency vis-a-vis every level of the health system. It also strengthens implementation science frameworks such as the Health Equity Implementation Framework (HEIF) that focus on contextual factors that influence health equity. Rather than focusing on end users choosing whether to participate in an intervention or not, health systems can be crafted to create cultural safety on each level of the system.

Even for women who give birth in health facilities, many do not access subsequent PNC visits. Barriers to PNC include being single versus being married, not being informed about PNC services, and experiencing overall health and not perceiving oneself to be in need of medical care.^27,28^ Facilitators for Ethiopian women receiving PNC include counseling from health providers, delivering at a health facility, having a Cesarean delivery, experiencing delivery complications, possessing an awareness of maternal health complications, outcome of birth (live versus stillbirth), and having a higher level of education.^21,27–30^ Higher socioeconomic status is also strongly associated with receiving one PNC visit; however, no correlation exists between higher socioeconomic status and more PNC visits.^31^ The availability of and relationship between HEWs and the community are another mediator to women receiving PNC. Differences in expectations between job description and performance, high workload, and breakdown of PNC referral system all contributed to these barriers.^18,19,32,33^

While PNC conducted in health centers and hospitals is performed by clinicians, PNC that takes place at home is managed by HEWs.^19^ One study found that 39.4% of those who received care at home were checked for heavy bleeding, 31.8% were counseled about breastfeeding, and 20% were counseled regarding family planning.^19^ The coverage is lower than care received in other LMICs at home visits.^19^

Similarly, women’s communities sometimes hide births so that religious-cultural rituals can take place. Beyond this, logistical issues such as lack of telephone often makes such contacts between HEWs and postpartum mothers difficult.^18^

Current constructs of individual use of postnatal services cannot account for the societal and economic and sociopolitical forces deeply imbricated in all aspects of maternal–child care. This study employed abductive analysis using the HEIF^34^ to reexamine barriers to implementation in Ethiopian PNC.^32,35^ This approach adds complexity to perceptions of maternal decision-making. We previously found that patients exercise agency to protect their cultural safety during the postnatal period.^22,36^ However, discussions of agency are framed as opting in or out of interventions rather than as embedded in each level of the health systems. We now examine how preservation of cultural safety influenced the use of PNC among women in Ethiopia. Using the HEIF, we conducted an abductive analysis of data from interviews with participants who sought to maintain their physical, spiritual, and emotional safety.

## Materials and methods

### Study setting

Ethiopia has four levels of administrative divisions: regions, zones, woredas, and kebeles.^3^ Each *woreda* has a primary hospital and five health centers on average. A *kebele* is the smallest government health unit, which has one health post. The Ethiopian Ministry of Health assigns 2 HEWs to each *kebele* and its health post, which serves 5000 people.^32,35,37^ The study was performed in three regions of Ethiopia: Afar, Oromia, and Southern Nations, Nationalities, and Peoples’ Region (SNNPR) among women who had given birth at hospitals and health centers. The team used delivery registries to identify participants. Study staff and local health staff helped the team purposively identify one hospital and one health center in each woreda that best represented the staffing, patient population, and service quality of each catchment area. Five women between the ages of 18 and 45 who had given birth in a government health facility between the previous 2 and 6 months were purposively selected per health center and hospital to reflect parity and age diversity

### Study design and sampling

This study used data collected as part of an earlier study that evaluated a district-wide health systems quality improvement intervention in Ethiopia by the Institute for Healthcare Improvement and the Ethiopian Ministry of Health using mixed methods.^38^ Semi-structured, in-depth interviews were conducted with women between March and April 2018 and in April 2019. The study team and health leaders at each study site collaborated to purposively select one hospital and one health center in each woreda that was representative of the birthing experiences in the catchment area.

#### Sample and sampling technique

The sample included 63 women who had recently given birth in three Ethiopian regions: SNNPR, Oromia, and Afar regarding their experiences receiving health care during recent deliveries. Participants gave verbal informed consent to participate in audio-recorded interviews. Interviews were then conducted six female research assistants (RAs). Two RAs worked in each of the three regions and were selected because of shared language and ethnic identity of participants. The semi-structured interview guide focused on participants’ most recent pregnancy and delivery. Open-ended questions were informed by the Donabedian Framework and included elicited information on staff interactions, services, and facility.^39^ The team used delivery registries to identify participants. Study staff and local health staff helped the team purposively identify one hospital and one health center in each woreda that best represented the staffing, patient population, and service quality of the catchment area. Five women between the ages of 18 and 45 who had given birth in a government health facility between the previous 2 and 6 months were purposively selected per health center and hospital to reflect parity and age diversity. Interviews took place in and near participants’ homes or other private areas. We use pseudonyms throughout the text to refer to participants.

#### Data collection and procedure

The interview process was iterative. RAs produced field notes that reflected their immediate reflections on interviews and regularly debriefed with the study team following each interview. Field notes were used to refine the interview guide for future interviews to delve deeper into topics emerging in the data and achieve concept saturation.^40^ The interviews ranged in duration between 30 and 90 min. The RAs sought private areas in which to interview the participants, such as inside of a parked car belonging to a member of the research team, an open field, or in the participants’ homes in order to ensure participant privacy. They were audio-recorded, and transcribed verbatim in the language in which they were conducted. Interviews conducted in Amharic were translated to English for analysis by the RAs. Interviews conducted in Afan Oromo, Afar, Dorze, Gamo, and Wolayetegna were first translated to Amharic before being translated to English. All RAs had one initial week-long field training, followed by analytic training which included data transcription.

#### Ethical consideration

The University of North Carolina at Chapel Hill’s Institutional Review Board and Ethiopian Public Health Association approved this research. Boards deemed the research program evaluation, and thus, the project was deemed exempt.

### Data analysis

**Figure 1.**
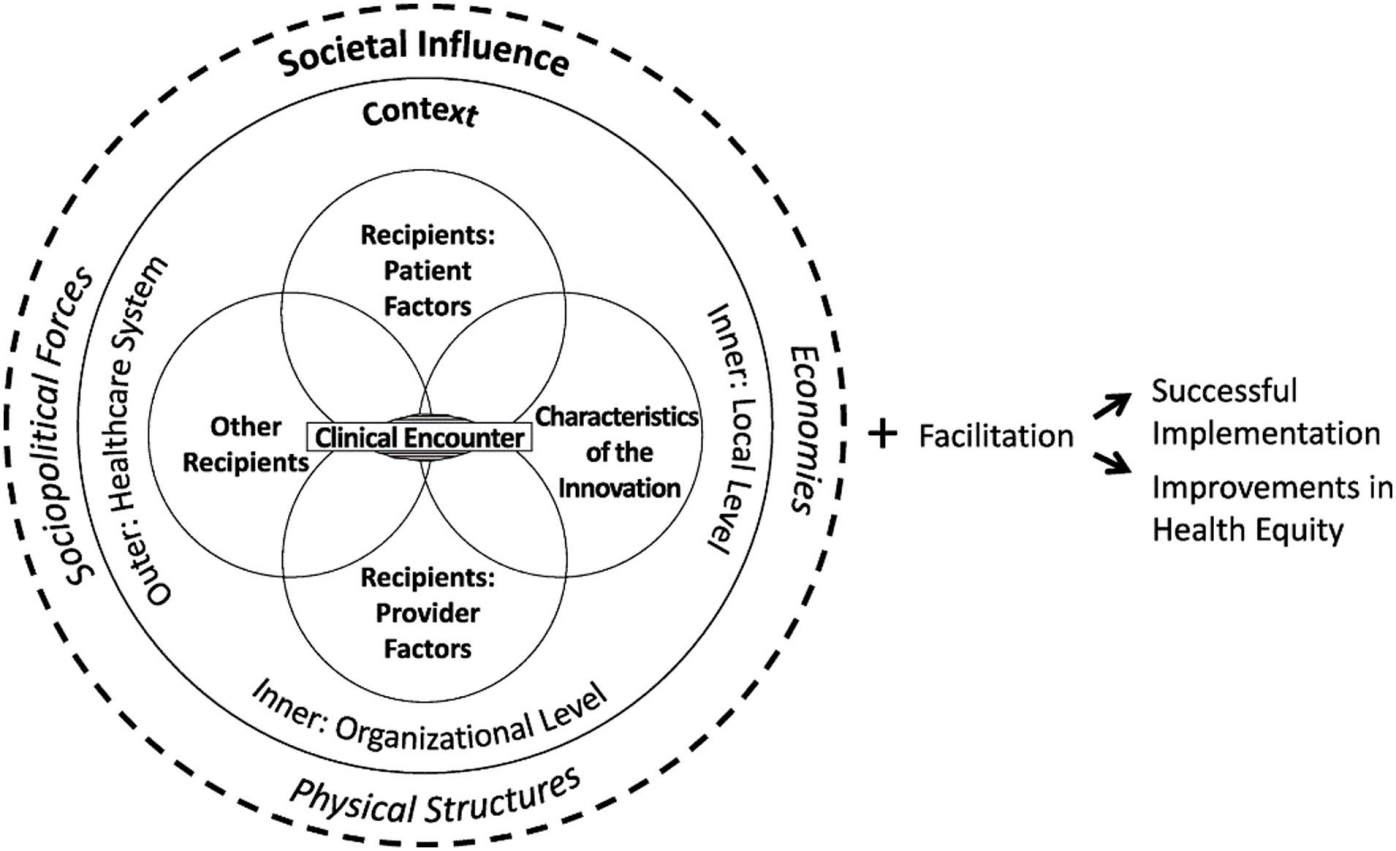
Health Equity Implementation Framework.

Our study is an abductive analysis of data from interviews with participants and the HEIF. The HEIF was developed as a combination of the Integrated-Promoting Action on Research Implementation in Health Services (i-PARIHS) framework^41^ and the Health Care Disparities Framework^42^ and “explains factors relevant to implementation and disparities in healthcare.”^34^ HEIF is represented in Figure 1. We chose HEIF as our abductive analytic anchor because it proposes that investigation of societal influence should be integrated into analysis of all implementation factors, rather than being reserved primarily to the outer context.^34^ HEIF proposed three areas of focus related to health equity that could be added to any determinant framework: attention to the clinical encounter, societal influence on every determinant, and health equity–specific recipient factors.^43^

HEIF centers the clinical encounter as the point at which the innovation is delivered. The clinical encounter is characterized as an interaction between recipients and the innovation. The inner context is comprised of both the local and organizational levels, which correspond to the clinic and hospital or network, respectively. The outer level is defined as the healthcare system. Finally, the model considers societal influence on innovations through sociopolitical forces, physical structures, and economies.

We took an abductive approach to analyzing interview data, using the codebook developed by the study team at the time of the first study and HEIF.^44^ Abductive analysis is an approach to qualitative research that prioritizes the construction of theory through methodically analyzing the relationship between “anomalous and surprising empirical findings” and extant theory.^45^ We chose an abductive approach because we recognize HEIF as a useful framework to organize and understand our primary data. At the same time, we observed that our data could add to the framework.^44^ The codebook was developed through regular debriefs and meetings to discuss interview, transcripts, and code production. It included metacodes reflecting context, healthcare experience, phase of care, relationships, satisfaction, and equity and disparities and codes within each of these categories. Transcripts were originally coded using ATLAS.ti and, later, NVivo when the research team changed institutions.^46^ A three-person coding team open coded 20% of the transcripts and met to perform line-by-line checks. They discussed each coding discrepancy and revised the codebook accordingly. The team double-coded half of the transcripts, met to resolve coding discrepancies, and finalized code definitions when there were no longer disagreements between team members regarding codes. The remaining transcripts were single-coded. We applied deductive codes to participants’ narration of their experiences using the Donabedian quality care framework.^39,47^ Inductive codes were developed through in vivo coding, idioms and euphemisms, and repetition-based theme analysis. Structural codes such as age group of participant, ethnicity, and birth facility type were also applied.

In our abductive analysis, the team first returned to portions of the data that were coded for PNC. This code described mothers’ experiences in receiving or not receiving healthcare services after delivery at health facility, home, or community setting. We then used NVivo to identify overlap between the structural PNC code and deductive and inductive codes to more deeply understand why implementation of the Ethiopian PNC program has not been successful. Overlapping codes were then grouped using an abductive approach using the domains of the HEIF and arranged as facilitators and barriers to accessing the clinical intervention of PNC. We chose an abductive analysis to leverage the strengths of both our deductive analysis based in the HEIF and our qualitative data. This theory-building approach highlights the interplay between observations and theoretical frameworks.^44^

The following is an example of our analytical process: Where the codes PNC overlapped with discrimination/prejudice code and where PNC overlapped with structural barriers, we mapped these codes onto “sociopolitical forces” in the HEIF using an abductive approach.

### Reflexivity statement

Our research team included Ethiopian and American academics with backgrounds in the history of medicine, public health, and anthropology, Ethiopian medical providers and public health professionals, and Ethiopian public health researchers. While all of us were outsiders with distance to our participants, our varying backgrounds generated different approaches to our data and this project. The RAs who collected our data had more in common with our participants in terms of life experiences and therefore were able to understand some cultural nuances. All coauthors are committed to understanding issues affecting access to maternal health services, and many including the two white American academics who led the analysis have worked in diverse settings. We remained close to our data and consulted our in-country research team when we needed clarification. The research team used memos and field notes to track their analysis over time, observe their relationship to the data, and identify questions that could be presented to the research team or that could be iteratively addressed by returning to the data. We consider our project a collaborative contribution to discussions of PNC use, individual agency, and implementation science, rather than an objective statement of fact.

### Reporting guidelines

We followed guidelines put forward in COnsolidated criteria for REporting Qualitative research (COREQ).^48^

## Results

We anchor our findings around utilization of PNC among Ethiopian women while considering their preservation of cultural and physical safety as an expression of agency and as critical to their care. We will describe the findings by domain, which are included in the subtitle of each section.

### Clinical encounter

#### Recipients: patient factors – culturally safe foundations

Patients were imbricated in culturally safe systems of traditions and religion that they trusted more than the care of the health system. Their prioritization of these systems over PNC within the formal health system were an expression of agency in order to maintain cultural safety. Table 1 shows the PNC codes as they correlate with HEIF domains and subdomains.

**Table 1.** Matrix of barriers: themes relating to the HEIF clinical encounter.

HEIF domain	HEIF subdomain	PNC codes	Health action or factor	Facilitated or impeded PNC
1. Clinical encounter	1a. Recipients: patient factors	Healthcare expectations	Was informed	Facilitated
			Was not informed	Impeded
		Feelings	Happiness of family members for using health care	Facilitated
		Traditions and customs	Staying home 40 days after birth	Impeded
			“Everything in God’s hands”	Impeded
			Not feeling bound to traditions and customs	Facilitated
			Postpartum health issue	Facilitated
			Baby health issues	Facilitated
	1b. Other recipients: family members, other participants in the EBI, birth support person	Family members	Family-supporting facility use	Facilitated
	1c. Recipients: Provider factors:	Trust	Trusting HEW	Facilitated
	1d. Characteristics of the Intervention		Dyadic care; HEW visiting home	Facilitated

HEIF: health equity implementation framework; PNC: postnatal care; EBI: evidence-based intervention; HEW: health extension worker.

Women expressed their care preferences within a social structure that they saw as critical to their well-being and a belief structure that they saw as having ultimate control over their lives. While this did not conclusively preclude them seeking care for medical appointments, they expressed a preference for staying in their homes following birth. Aleke, an Orthodox Christian woman from SNNPR, reflected on the custom of not leaving one’s house for several weeks following birth. When asked when she left her bed to resume daily life following her baby’s birth, she responded: “I am not out yet; you have to wait 80 days for a baby girl and 40 days for a boy . . .” (26 years, two live births, Orthodox, SNNPR). Although she had an appointment scheduled for the next day, she affirmed that she did not want to go. Chaltu, an Orthodox woman from Oromia, elaborated on this custom, called Christina: “After delivery a woman is not allowed to work at home until she go to church and pray. She is not allowed to enter to the kitchen until that. There is a ceremony called ‘Christina’ which means simply giving baby to God. It is held after 80 days, until then the delivered woman shouldn’t work outside. This is what religiously not allowed” (35 years, three live births, Orthodox, Oromia). While illness would be considered an exception to this custom, Chaltu said that, thanks to God, she had remained well and therefore continued to remain in her house. After Christina had taken place, she affirmed that she “could go everywhere,” as well as return to her domestic duties.

Regardless of religious customs that interfered with the logistics of seeking care, many women reported that they believed the outcome of the pregnancy and birth processes were in God’s hands and did not perceive themselves as playing a decisive role in the outcomes of their pregnancies and births. Ebise explained that part of the welcoming ceremony for her and her baby upon returning from the health center was thanking God for the safe delivery of her baby. The welcome ceremonies underscored the connection between community support and cultural custom. She explained, “According to our culture, coffee ceremony was prepared; injera was baked; everything was ready, people brought coffee, they hugged and kissed me saying ‘congratulations!’ Thanks to God for separating you from your offspring safely.” (40 years, four live births, Orthodox, Oromia). The cultural customs for the postpartum period were deeply imbricated in a religious context of God’s will regarding the well-being of mothers and babies in the weeks following birth.

While the women in our study delivered in health centers and hospitals and many said they would seek future medical care when necessary, most said they considered their health to ultimately be in God’s hands. When asked about her expectations about future pregnancies, Jemila answered, “that God gives the child” (18 years, one live birth, Muslim, Afar). Another participant, Kimia, explained that medical intervention could only go so far: “What will they give me only the blood they give me and the drugs they do and lastly its God who helped me to be healthy.” When asked about the role of health workers, Kimia continued, “Yes it was God’s will but they help me from their side and nothing left from the things they do if God doesn’t will I would have been sick and not healthy. . . . . you see. . . so God helped me with it.” (20 years, one live birth, Muslim, Afar). Kimia’s responses reflect how participants described an external locus of control in which their actions played only a small part in the safety of them and their babies during this period. Kimia’s experience highlights how women often did not seek PNC because they considered themselves to be well. As Kimia explained, because she did not perceive symptoms of unwellness during the postnatal period, she deemed herself well and that God was maintaining her health.

In these three Ethiopian regions, many women appreciated the benefits of health care and health providers but perceived their benefits to be an extension of God’s will. Community support provided assistance that was consistent with these religious beliefs. When women did leave their homes to seek care, it was usually for a clearly adverse health event, such as excessive bleeding.

#### Recipients: provider factors – pursuing useful care

Women were more inclined to receive care when the HEW visited their homes, because they could satisfy care recommendations without leaving their support system. A home visit was more likely to make them deem PNC important. When asked whether she made an appointment for PNC at the hospital Dalbore explained “No, I don’t have an appointment . . . The health care provider came to my home and immunizes the baby and check up on my health status” (30 years, three live births, Protestant, SNNPR).

Moreover, when they built trust with their HEW, women described following through with care, as well as developing trust in the health system generally. When asked about her relationship between her HEW and mothers in her community, Dalgite responded that the HEW’s consistency had led to a positive relationship in which the mothers received support, ongoing education, and health care such as vaccines. She described how her HEW provided her baby vaccines, gave her breastfeeding support and health information regarding her pregnancy, as well as her confidence in the HEW. She also expressed confidence in the education that the HEW provided: “I know the education is a good thing for my baby” (30 years, three live births, Protestant, SNNPR). When HEW care was consistent and deemed useful by participants, they were receptive to receiving it.

#### Characteristics of the intervention: importance of regular contact and home contact

Some women who received PNC reported that it was convenient to do so when the HEW came to their homes and when they performed care for their babies at the same time. The model of dyadic care for mother and baby, as well as an overall confidence in vaccination that participants expressed, made receiving care more culturally safe for them. When HEWs were able to visit homes, women could access care, as well as care for their newborns while maintaining cultural safety. Moreover, regular contact with HEWs built trust between them and the community and made women more inclined and more able to access such care when it was available to them. Dalgite explained of her HEW, “She comes every day whether she has a vaccine appointment or not.” While women did not commonly report that HEWs visited them every day and HEW visits varied depending on availability and need, several who utilized PNC received it through regular visits in their homes.

### Inner context: lack of organizational and system communication

Our findings show that many women did not attend a PNC visit because they were not told such a visit was necessary. Overwhelmingly, our findings suggest participants were not made aware of the necessity of PNC.

#### Inner context: local level – lack of communication

Women’s agency was limited by not knowing about the importance of or recommendation for PNC appointments due to a lack of communication between the health facility and themselves, as well as the health facility and the HEW. Table 2 shows the PNC codes as they correlate with HEIF domains and subdomains as related to inner context. Women across all three regions reported that no one told them they needed to schedule a PNC appointment and or asked them to schedule one. Maryam explained, “They didn’t tell me so I didn’t know; they didn’t say anything about visit experience or practice after delivery . . .” (20 years, four live births, Afar, Muslim). Others participants said they were told to come back only if they experienced an obvious health issue, such as bleeding. Some, such as Kimia, sought care for health conditions and said they only went because of these issues (24 years, four live births, Afar, Muslim). Table 3 depicts responses from each respondent who reported that she was not told about the need for PNC.

**Table 2.** Matrix of barriers: themes relating to HEIF inner context.

HEIF domain	HEIF subdomain	PNC codes	Health action or factor	Facilitated or impeded PNC
2. Context factors Inner context: local level	2a. Inner organizational level	Facility delivery	Received inadequate care	Impeded
			Did not want to leave baby	Impeded
	2b. Inner local level	Postnatal care	Never told to return	Impeded
	2c. Outer healthcare system		Consistent HEW visits	Facilitated

HEIF: health equity implementation framework; PNC: postnatal care; HEW: health extension worker.

**Table 3.** Participant responses to being asked whether they were told about the importance of PNC.^a^.

IDI	Response to being asked about PNC
IDI 02	“I don’t know that . do we need to go even if my baby is doing well.”
IDI 04	“They *only* told me that there is vaccination for me and my baby after 45 days.”
IDI 06	“After delivery they didn’t tell me to come back.”
IDI 07	Was told to visit, “if your children are sick and something wrong with them. . . and after two days of delivery if your blood didn’t stop.”
IDI 08	“No, no one give me appointment *after delivery*”
IDI 10	“Nobody told me something like that” *to come after delivery*
IDI 12	“Interviewer: no one counselled or appoint you after you gave birth to come back for postnatal care?: P: “Yes”
IDI 16	“No one gave me appointment. I went to my house after one day.”
IDI 17	“What kind of follow – up?… we *went* to the center to vaccinate the baby.”
IDI 18	Interviewer: “no one gave you information or counselling to come after giving birth for post natal care follow up? Participant: “yes”
IDI 27:36	“They didn’t say anything, they just *discharged me*.”
IDI 27:35	“I didn’t learn about that after delivery.”
IDI 39:69	“I didn’t have an appointment.”
IDI 27:98	“They didn’t say anything, they just told me to go out.”
IDI 39: 77	“They did not appointed me.”
IDI 42:#	“I did not get an appointment.”

PNC: postnatal care.

a Italics are used to note paraphrasing.

The above findings underscore that women exercised their agency in accessing care when they were adequately communicated with and regarded that care as necessary.

#### Inner context – organizational level: insufficient care networks in system

Some participants reported that the clinical and hospital network did not meet women’s needs for care for their babies. Some women reported seeking PNC would require them to leave their babies at home, something they did not want to do. One participant declined to leave her home despite experiencing an obvious health issue, because she could not take her baby. She explained, “I ask them what should be done if there is bleeding? And they told me that they will give medication and told me to come back after 6 weeks. But I did not go. . .I was thinking about how could I go holding my baby, So I did not go.” Fatuma, from Afar, explained that she lost trust in the system because staff could not accommodate her delivery because she had experienced female genital cutting (FGC). The staff was surprised when her baby was born so quickly because women who have experienced FGC usually require staff intervention to delivery their babies. A male nurse rushed in to deliver the baby. When asked what kind of care she expected and received from the staff, she responded, “I didn’t expect anything from them . . . . .nothing. My mom was the one who did everything and she did whatever I want.” (20 years, one live birth, Afar, Muslim). After her delivery, a male nurse inserted his hand to stop bleeding, even though Fatuma had requested that a male nurse not touch her due to her religious beliefs. Because of the lack of culturally safe care she received, Fatuma later lied to the nurse when he asked whether she had stopped bleeding and subsequently left the hospital before she could receive adequate PNC to address her bleeding issue. These findings on the organizational level build on those from the local level and explicitly highlight limitations of understanding the use of PNC within the Ethiopian health system as a choice made by individuals.

#### Outer healthcare system: self-protection after mistreatment

Some women’s previous healthcare experiences influenced whether they chose to seek additional care. Maryam expressed that she had lost trust in the health center after the inadequate care she received during her second pregnancy. “In previous times they checked us to see if something was wrong. They told us to come to the center if we felt dizziness. I even checked myself without my appointment. They took every examination, if there were symptoms they treated us immediately. This recent one I don’t know what they did for me.” (20 years, four live births, Afar, Muslim). Her dissatisfaction extended to the care her baby had received. Referring to a health visit they had attended together, she explained: “They didn’t watch him properly,” she explained. “they just gave us syrup. I gave 30 birr (Ethiopian currency) for them to come back to my house.” She went on to clarify that she wanted to seek care for herself, but would not do it in the health center where she had been mistreated. “I like to be treated by health care,” she said. “I don’t think I will stop myself from going there and things happen with GOD’s will. But I don’t go to [the health center]for delivery.” She explained that in addition to lack of care, she had been mistreated during birth by being attended to by a male provider, which went against her religious beliefs and because staff did not know how to deliver her baby due to her scar from FGC. Although she was experiencing an illness during the interview, she was not motivated to visit the health center until after her 45 days: “What will they do if we go to them?”

For Maryam, seeking PNC was not worth the physical or financial risk to her and to her baby. Her story emphasizes how women made active choices to protect themselves in situations in which the health system did not provide adequate or safe care for themselves and their babies. Their experiences within the health system, their desire for self-preservation, and the difficulties of navigating the insecurities of the health system strongly influenced women’s willingness and ability to continue care within the health system and influenced their choices around receiving such care.

#### Outer context: healthcare system (formal and informal)

Women negotiated faulty health systems and logistical issues to receiving care and often perceived themselves to be best supported in their home communities, which facilitated practical care, as well as their cultural requirements. Table 4 shows the PNC codes as they correlate with HEIF domains and subdomains.

**Table 4.** Matrix of barriers: themes relating to HEIF outer context.

HEIF domain	HEIF subdomain	PNC implementation theme	Health action or factor	Facilitated or impeded PNC
1. Societal context	3a. Sociopolitical forces	Vaccines	Infant vaccines	Facilitated
			Maternal vaccines	Facilitated
	3b. Economies	Finances	Not having enough money for appointment	Impeded

HEIF: health equity implementation framework; PNC: postnatal care.

#### Sociopolitical forces: perceived necessity of care

Women took more action to receive health care that they perceived to be important for themselves and their children. Vaccine recommendations for both infants and mothers were an effective motivator for women following up on PNC recommendations. Lechame described how her social network enabled her to stay home with her baby for 3 months after her birth, but vaccination prompted her to leave her compound. She explained, “They stay for 4 months or so. . . for me it has been 3 months and I have not left the compound yet.” Interviewer: “What about for vaccinations and your follow-up?” Lechame: “For that I will go, I did go. . .I went for follow-up on the 5th day. For vaccination I went one day with the baby.” (28 years, two live births, Protestant, SNNPR). This was a concrete health intervention Lechame understood she needed, versus a recommendation for PNC they perhaps did not think they needed because of their relative health.

Family members and neighbors deeply influenced women’s daily lives in that many were cared for in their homes and did not seek help elsewhere and because of deeply embedded social traditions around birth. These social networks functioned as an informal care network that in some ways replaced the formal health system.

#### Finances

Several participants described complex risk weighing around the value of health visits versus the resources these visits would cost them. Halima described accessing health care as contingent on her finances, which were especially stressed after she lost some of her money on the way to a hospital in Addis Ababa to seek care for her baby: “When we entered [the hospital] the doctors asked me if I had money. . . I told them I have enough money and he can see my account from the bag . . .he said yes you have enough. Thanks to God, if I couldn’t have the account book I don’t know what will happen because I lost the 30 thousand birr” (26 years, five live births, Muslim, Afar). Others, like Rahima, said even when experiencing an obvious health issue she had been warned about, they did not go to the health center because of lack of financial means. When asked why she did not take her baby to the health center, she responded, “I was out of money and my focus was on my baby. . . transportation is very problematic to go to Berta and you have to walk a far distance to reach the health center” (20 years, one live birth, Protestant, Afar). However, for women to whom PNC valued a visit, they worked hard to overcome barriers to receiving it.

## Discussion

Our study addressed the set of goals developed by the Ethiopian Ministry of Health to increase demand for PNC by 2025 (RH Strategic Plan).^7,32,35^ We employed an abductive analysis using our inductive–deductive codebook and the HEIF to identify novel understandings of end users interfacing with PNC in Ethiopia.^49,50^ Our contributions converge around how understanding that patients seek to maintain their cultural safety reframes many so-called culture-based barriers as a continuous expression of agency.

By presenting patients as acting to preserve their cultural safety, our paper adds important complexity to implementation research literature on health equity.^43,51,52^ The HEIF draws focus to community and cultural factors that influence individual choices and adherence to health protocols.^34,42^ With its requirement that patients must determine whether cultural safety is met, it responds to the emerging standard of engaging end users in health equity research,^23,53^ which early evidence suggests can be useful in increasing health behaviors and improving outcomes.^54^ Approaching end users as maintaining their safety during health encounters also speaks to the goals of implementation science to tailor interventions to a specific community,^55,56^ reflexivity on behalf of health systems and scholars in implementation science,^57^ and more standardized methods for increasing end user engagement,^58,59^ while acknowledging that end users are imbricated within complex contexts.

**Figure 2.**
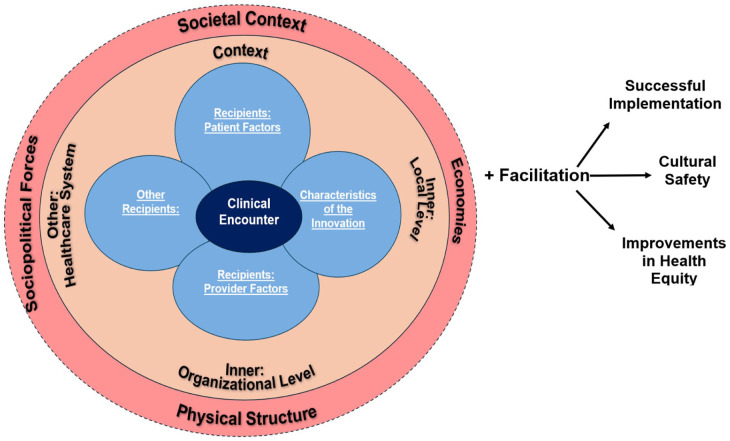
Health equity implementation framework with proposed cultural safety addition.

We propose the addition of a process outcome of cultural safety to HEIF to highlight its role in increasing service utilization and thereby increasing positive health outcomes, as portrayed in Figure 2. Studies have already noted that cultural safety leads to better health outcomes in LMICs.^60–63^ This study joins emerging scholarship that focuses on quality of care in the wake of the World Health Organization’s call for an emphasis on this theme.^2,36,47^ When women perceived care as important, they took steps to access it. For example, many women understood vaccination as an important intervention both for them and for their babies and actively pursued this health care even when it required temporarily breaking with their customs. Implementation strategies must therefore account for how patients frame trust within health systems, which influences how women make their decisions about their health when proposing educational interventions.

Maternal health researchers cite postpartum care as a top priority for implementation research.^64^ However, maternal–child health interventions remain underrepresented in implementation research and most literature has focused on facilitators and barriers to care, rather than applying theories, models, and frameworks.^65^ Furthermore, while some of these theories, models, and frameworks examine the aspects of context that contribute to individual adherence to health interventions, the roles of culture and societal influence in people’s adherence remain largely underexamined.^66,67^ Of the few implementation science interventions in maternal–child health papers, several have cited determinant frameworks, such as the Consolidated Framework for Implementation Research (CFIR). The CFIR 2.0 Framework includes a societal pressure domain that is divided between roles and characteristics.^67^ HEIF allows for further breaking down of the individual domain with focus on patients, providers, other recipients, and the clinical encounter and places these encounters between individuals at the center of analysis.^34^ Our inclusion of cultural safety as a process outcome within HEIF extends this literature by addressing individual barriers and facilitators to receiving care as imbricated in a larger structure, rather than as separate domains.

Our article also joins recent implementation research in LMICs that has pointed to context-specific needs in the education and training of healthcare workers^68,69^ and the need for respectful maternity care in LMICs ^70,71^ framed by the Ethiopian PNC crisis.^20,21^ Satisfaction with care is increasingly recognized as a necessity for quality care.^51^ The inclusion of cultural safety provides the means for achieving cultural respect across contexts with its principles of ongoing provider reflexivity,^23,72^ rather than a prescribed list of requirements for respectful care.^26,73^ For example, previous literature has cited many culture-based reasons that women do not seek PNC in Ethiopia, which include the influence of local religious leaders and religious ceremonies that favored women giving birth at home or leaving the health facility before receiving or organizing future PNC and the belief that religious support rather than health care will be more efficacious for preventing complications during the postpartum period.^16,18,19,32,33,35^

Cultural safety also provides a way of thinking through the “black box” of matching implementation strategies onto barriers to care.^74^ A review of perinatal participants’ understanding of culturally safe interventions highlights birthing in community, acknowledgment of difference, and respect for culturally situated knowledge.^26^ This emerging knowledge of cultural safety’s use in health encounters allows us to map implementation strategies onto HEIF more clearly.^75^ Creating learning collaborative^75^ addresses need for acknowledging difference and respecting end user’s knowledge.^26^ Involving patients and family members^75^ addresses need to experience health care in community. Obtaining and using user and family feedback^75^ explicitly references cultural safety’s requirement that patients decide whether cultural safety has been achieved^23^

### Study recommendations

Several validated tools for evaluating and implementing culturally safe care exist that have been tested in HICs. Future studies could test strategies that have been successful in HICs such as training providers and administrators in skills such as active listening^76^ in LMICs. Additionally, future studies could test assessment of cultural safety^77^ and evaluation of cultural safety programs in LMICs.^78–80^ This will add to calls for clear clinical definitions of cultural safety so that interventions can be more culturally safe.^81^

Utilizing these tools at every level of the health system will takes emphasis off patients, who, as nonmedical experts, cannot be expected to state their health needs thoroughly. This systems-level approach also takes emphasis off hyper-burdened providers and the clinical encounter:^82^ Training of clinicians in cultural competency has had so far limited or poor effects^83–85^ Rather, an approach to cultural safety must be integrated into the whole health system in order to effectively engage end users in their many contexts.

### Strengths and limitations

Our study has limitations. First, because we only sampled from three Ethiopian regions, our study may not be transferable to women in all regions of Ethiopia. Linguistic nuances could have been lost in the process of doubly transcribing interview audio into English. We asked women to describe their prenatal, birth, and postnatal experiences, many of which included intense [things]; this could have led to women feeling uncomfortable sharing, despite rapport building and establishing consent on behalf of the interviewers. Because inductive analysis relies on an interaction between deductive and inductive information, it is limited by the theories and constructs involved in the analysis. In this case, our results are both supported and limited by the HEIF. The limitations of abductive analysis, similar to deductive and inductive methodologies, are related to the available data and analytical frameworks. Moreover, some scholars have critiqued the methodology as not being distinct from induction and leading to confirmatory hypotheses that cannot be tested.^86^

## Conclusions

Our analysis provided insight as to how social, cultural, and structural factors overlap influence women’s decisions to seek care during the postpartum period. Our findings indicate that women’s agency guides their decision-making based on how they perceive their well-being to maintain their commitment to cultural and religious practices, obtain support within their community, and to limit potentially adverse healthcare experiences. Utilization of a HEIF ensures that these multiple and overlapping factors address the importance of social place and agency when mothers make decisions about PNC.

## Supplemental Material

sj-docx-1-whe-10.1177_17455057261424102 – Supplemental material for “God gives the child”: An abductive analysis of barriers to postnatal care using the Health Equity Implementation Framework Supplemental material, sj-docx-1-whe-10.1177_17455057261424102 for “God gives the child”: An abductive analysis of barriers to postnatal care using the Health Equity Implementation Framework by Emilie Egger, Befikadu Bitewulign, Humberto Gonzalez Rodriguez, Haley Case, Abiyou Kiflie Alemayehu, Elizabeth C. Rhodes, Abiy Seifu Estifanos, Kavita Singh, Dorka Woldesenbet Keraga, Marukh Zahid, Hema Magge, Dara Gleeson, Clare Barrington and Ashley Hagaman in Women's Health
