# Supplementary material for: “God gives the child”: An abductive analysis of barriers to postnatal care using the Health Equity Implementation Framework
Source: Womens Health (Lond). 2026 Apr 6;22:17455057261424102. doi: 10.1177/17455057261424102 (PMC13053962; doi:10.1177/17455057261424102)
Supplement: sj-docx-1-whe-10.1177_17455057261424102 – Supplemental material for “God gives the child”: An abductive analysis of barriers to postnatal care using the Health Equity Implementation Framework [file sj-docx-1-whe-10.1177_17455057261424102.docx]

**Participant ID**: ______________

**Date:** _____________________

**Interviewer name:** _________________________

**Language of Interview:** _____________________

**Translator (if present):** ___________________

**Region**: _____________________

**Woreda**: _____________________

**Health facility name:** ______________________

What is your age? ______

How many live births have you had? ________

How many of your children are alive? ______________

How old were you when you gave birth to your first child? _______

What is your marital status? _______________ What is your ethnicity? ____________________

What is your occupation? ______________________ What is your religion? _______________

How many people eat from the same plate in your house? ___________

What is the highest school grade you completed? ____________

Can you read? ______ Can you write? _________

How many times did you visit this clinic during your pregnancy, including delivery? ______

Did you receive care at other health centers during your pregnancy? ____Yes ____No

If yes, which health center? ________________________________

What was the reason for getting care from another health center? ________________________

PURPOSE: build rapport

1. To begin, I’d like to know about you. Tell me, what is it like for you being a mother?

- Tell me about your family.

PURPOSE: understand pregnancy experience

1. Tell me about your most recent pregnancy, how did you find out you were pregnant?

- Describe some of the emotions you felt throughout your pregnancy.

1. Who gave you advice during your pregnancy?
   - What did people tell you?
   - Who were these people?

PURPOSE: understand prenatal care experience

1. I would now like to learn about your experiences with prenatal care (follow-up). Tell me, how you begin follow-up services at the health center/hospital?

- How often did you come for prenatal care?
- Who, if anyone, helped you get care at the facility?
- What, if any, concerns did you have coming to this clinic/facility? Tell me more…

1. Describe one follow-up (ANC) visit from the moment you arrived until you left the health center/hospital. What was this like?
2. How did health staff treat you during follow-up visits?

- Give me example of what they did.

1. Based on your follow-up (prenatal) visits, what did you expect for your delivery?

Purpose: understand delivery experience

1. Tell me about your delivery experience. Start from the moment you laboring until you left the health center/hospital after giving birth.
2. What did you think about your delivery experience at the health center/hospital?

- Why did you feel this way?

1. SPACE: I do not know much about the health center/hospital you attended. Can you describe the space where you received services to me?

- How would you describe the cleanliness? Privacy?
- What did you like? Tell me more…
- What did you dislike? Tell me more…

1. STAFF TREATMENT: Tell me about the staff at the health center/hospital. How did they treat you during your delivery? Tell me about this…

- Can you give me example?
- How did that make you feel?
- Who were these people?

1. How confident did you feel in the knowledge and skills of clinic staff? Tell me more…

PURPOSE: understand postal natal care experience

1. Now I would like to learn about your follow-up experiences after your baby was born. Did you receive any follow-up two days after delivery at the clinic or at your house? Tell me about this…
2. What kind of follow-up did you expect after your baby was born?

- Tell me more….
- Does your community have a confinement practice after delivery? Tell me about this…

PURPOSE: understand health satisfaction

1. How do you believe the health care you received helped you or your child?
2. Based on your experiences, would you use these services again? Tell me more about why or why not.
3. Would you recommend these services to other pregnant women? Tell me more about why or why not…
4. How can the health center/hospital make the experience of childbirth better for women such as yourself?

- What is needed?
- Who should do this?

1. Thank you for sharing your experiences with me, I really appreciate your time.

- Do you have any questions or comments for me?
